# Association of respiratory symptoms and lung function with occupation in the multinational Burden of Obstructive Lung Disease (BOLD) study

**DOI:** 10.1183/13993003.00469-2022

**Published:** 2023-01-12

**Authors:** Jate Ratanachina, Andre F.S. Amaral, Sara De Matteis, Herve Lawin, Kevin Mortimer, Daniel O. Obaseki, Imed Harrabi, Meriam Denguezli, Emiel F.M. Wouters, Christer Janson, Rune Nielsen, Amund Gulsvik, Hamid Hacene Cherkaski, Filip Mejza, Padukudru Anand Mahesh, Asma Elsony, Rana Ahmed, Wan Tan, Li Cher Loh, Abdul Rashid, Michael Studnicka, Asaad A. Nafees, Terence Seemungal, Althea Aquart-Stewart, Mohammed Al Ghobain, Jinping Zheng, Sanjay Juvekar, Sundeep Salvi, Rain Jogi, David Mannino, Thorarinn Gislason, A. Sonia Buist, Paul Cullinan, Peter Burney

**Affiliations:** 1 National Heart and Lung Institute, Imperial College London, London, UK; 2 Dept of Preventive and Social Medicine, King Chulalongkorn Memorial Hospital, The Thai Red Cross Society, Bangkok, Thailand; 3 Dept of Preventive and Social Medicine, Faculty of Medicine, Chulalongkorn University, Bangkok, Thailand; 4 Dept of Medical Sciences and Public Health, University of Cagliari, Cagliari, Italy; 5 Unit of Teaching and Research in Occupational and Environmental Health, Cotonou, Benin; 6 University of Cambridge, Cambridge, UK; 7 Liverpool University Hospitals NHS Foundation Trust, Liverpool, UK; 8 Obafemi Awolowo University, Ile-Ife, Nigeria; 9 Faculte de Medecine, Sousse, Tunisia; 10 Ludwig Boltzmann Institute for Lung Health, Vienna, Austria; 11 Maastricht University Medical Center, Maastricht, The Netherlands; 12 Respiratory, Allergy and Sleep Research, Dept of Medical Sciences, Uppsala University, Uppsala, Sweden; 13 Dept of Clinical Science, Faculty of Medicine, University of Bergen, Bergen, Norway; 14 Dept of Pneumology, Faculty of Medicine and CHU Annaba, Annaba, Algeria; 15 Center for Evidence Based Medicine, 2nd Department of Internal Medicine, Jagiellonian University Medical College, Kraków, Poland; 16 JSS Medical College, JSSAHER, Mysuru, India; 17 The Epidemiological Laboratory, Khartoum, Sudan; 18 Centre for Heart Lung Innovation, University of British Columbia, Vancouver, BC, Canada; 19 Royal College of Surgeons in Ireland and University College Dublin Malaysia Campus, Penang, Malaysia; 20 Dept of Pulmonary Medicine, Paracelsus Medical University, Salzburg, Austria; 21 Aga Khan University, Karachi, Pakistan; 22 University of the West Indies, St Augustine, Trinidad and Tobago; 23 University of the West Indies, Kingston, Jamaica; 24 College of Medicine, King Saud bin Abdulaziz University for Health Sciences, King Abdulaziz Medical City in Riyadh, Saudi Arabia; 25 State Key Laboratory of Respiratory Disease, National Clinical Research Center for Respiratory Diseases, Guangzhou Institute of Respiratory Health, First Affiliated Hospital of Guangzhou Medical College, Guangzhou, China; 26 Vadu Rural Health Program, KEM Hospital Research Centre Pune, Pune, India; 27 Pulmocare Research and Education Foundation, Pune, India; 28 Lung Clinic, Tartu University Hospital, Tartu, Estonia; 29 University of Kentucky, Lexington, KY, USA; 30 Dept of Sleep, Landspitali University Hospital, Reykjavik, Iceland; 31 Faculty of Medicine, University of Iceland, Reykjavik, Iceland; 32 Oregon Health and Science University, Portland, OR, USA

## Abstract

**Background:**

Chronic obstructive pulmonary disease has been associated with exposures in the workplace. We aimed to assess the association of respiratory symptoms and lung function with occupation in the Burden of Obstructive Lung Disease study.

**Methods:**

We analysed cross-sectional data from 28 823 adults (≥40 years) in 34 countries. We considered 11 occupations and grouped them by likelihood of exposure to organic dusts, inorganic dusts and fumes. The association of chronic cough, chronic phlegm, wheeze, dyspnoea, forced vital capacity (FVC) and forced expiratory volume in 1 s (FEV_1_)/FVC with occupation was assessed, per study site, using multivariable regression. These estimates were then meta-analysed. Sensitivity analyses explored differences between sexes and gross national income.

**Results:**

Overall, working in settings with potentially high exposure to dusts or fumes was associated with respiratory symptoms but not lung function differences. The most common occupation was farming. Compared to people not working in any of the 11 considered occupations, those who were farmers for ≥20 years were more likely to have chronic cough (OR 1.52, 95% CI 1.19–1.94), wheeze (OR 1.37, 95% CI 1.16–1.63) and dyspnoea (OR 1.83, 95% CI 1.53–2.20), but not lower FVC (β=0.02 L, 95% CI −0.02–0.06 L) or lower FEV_1_/FVC (β=0.04%, 95% CI −0.49–0.58%). Some findings differed by sex and gross national income.

**Conclusion:**

At a population level, the occupational exposures considered in this study do not appear to be major determinants of differences in lung function, although they are associated with more respiratory symptoms. Because not all work settings were included in this study, respiratory surveillance should still be encouraged among high-risk dusty and fume job workers, especially in low- and middle-income countries.

## Abstract 2

**Occupational exposures associate with more respiratory symptoms but do not appear to be major determinants of differences in lung function.** https://bit.ly/3zFjalu

## Introduction

Irreversible airflow obstruction often accompanied by dyspnoea, persistent cough and phlegm production are characteristic of chronic obstructive pulmonary disease (COPD). It has been estimated that workplace exposure may account for 10–18% of COPD cases in the population [1]. However, these estimates are based on studies whose designs, definitions of disease and exposure assessments are not easily comparable. In addition, most of the studies were undertaken in high-income countries (HICs) [2], and there is a relative lack of knowledge concerning low- and middle-income countries (LMICs), where farming and manufacturing under weak health and safety regulations remain common.

In this analysis, we assessed the association of respiratory symptoms (*i.e.* chronic cough, chronic phlegm, dyspnoea and wheeze) and lung function parameters (*i.e.* forced vital capacity (FVC) and forced expiratory volume in 1 s (FEV_1_)/FVC) with occupational exposures in the large multinational population-based Burden of Obstructive Lung Disease (BOLD) study, which collected data across several regions of the world in a standardised manner.

## Materials and methods

### Study participants

The BOLD study design and rationale have been described elsewhere [3]. Representative samples of at least 600 non-institutionalised adults, aged ≥40 years, were recruited from 41 sites in 34 countries (table 1) [4]. As classified by their gross national income per capita [5], 14 sites were in HICs and 27 sites in LMICs. Information on respiratory symptoms and exposure to potential risk factors, including occupation, was collected through face-to-face interviews conducted by trained and certified staff in the participant's native language. This report is based on data from 28 823 participants who completed the core and occupational questionnaires and provided acceptable and repeatable post-bronchodilator spirometry measurements. All sites received approval from their local ethics committee, and participants provided informed consent.

**TABLE 1:** Characteristics of participants from 41 sites of the BOLD Study with acceptable and repeatable post-bronchodilator spirometry and occupational exposure data

**BOLD site**	**Albania (Tirana)**	**Algeria (Annaba)**	**Australia (Sydney)**	**Austria (Salzburg)**	**Benin (Sèmè-Kpodji)**	**Cameroon (Limbe)**	**China (Guangzhou)**	**Canada (Vancouver)**	**England (London)**	**Estonia (Tartu)**	**Germany (Hannover)**	**Iceland (Reykjavik)**	**India (Kashmir)**	**India (Mumbai)**
**Subjects, n**	939	890	541	1253	698	331	461	827	675	613	680	757	760	439
**Age (year)**	54.6±10.8	52.5±9.9	58.9±12.4	57.7±11.4	51.5±9.8	51.3±9.9	54.0±10.6	56.0±11.8	58.2±11.5	60.9±12.0	58.1±11.0	56.4±11.7	51.4±10.4	51.1±8.9
**Height (cm)**	164.2±8.8	164.6±9.7	165.3±9.6	170.1±8.9	164.9±8.0	165.8±8.0	160.0±8.4	167.2±10.1	168.1±9.7	169.2±9.8	169.1±9.6	173.1±9.4	160.5±8.8	160.8±8.4
**BMI (kg·m^−2^)**	28.0±4.7	28.3±5.7	28.0±5.2	26.4±4.2	26.4±5.6	26.6±5.4	23.3±3.3	26.7±5.2	27.1±5.0	28.5±5.3	27.3±4.6	27.9±4.9	22.4±3.6	23.8±4.0
**Sex (male)**	49.7	49.7	49	54.5	43.3	59.5	49.7	41.6	47.6	50.2	51	53.2	54.7	62.6
**Never-smokers**	62.9	61.7	46	44.8	98	77.6	56.4	43.2	35.9	52.4	38.1	33.7	45.1	90.2
**<20 pack-years**	10.2	15.7	31.1	28.6	1.9	16.6	18.7	33.9	32.9	30.2	30.2	41	7.4	7.7
**≥20 pack-years**	26.8	22.6	22.9	26.7	0.1	5.7	25	23	31.3	31.8	31.8	25.4	47.5	2.1
**Chronic cough**	8.8	3.2	7	5.3	2.4	0.9	5.6	11.3	12.2	7	8.4	11.5	5.7	2.1
**Chronic phlegm**	1.8	2.6	5.7	7.9	2.2	1.2	6.9	10.6	11.7	9.4	8.2	9.3	5.7	2.3
**Wheeze**	3.7	14.5	25.4	13.2	2.8	4.5	1.5	26	34.2	22.8	18.7	24.2	3	3.2
**Dyspnoea**	8	11.8	7	6.6	1.4	5.8	3.8	6.9	12.1	14	4	8.4	4.9	9.9
**FEV_1_/FVC (%)**	78.4±9.0	78.6±7.3	76.4±8.9	74.3±8.6	79.3±7.1	80.4±6.9	78.1±7.3	76.0±8.8	75.0±9.2	77.2±7.8	76.2±7.9	76.1±8.5	76.4±10.6	79.1±7.5
**FVC (L)**	3.6±0.9	3.4±0.9	3.6±1.0	4.0±1.0	2.8±0.7	3.0±0.8	3.1±0.8	3.9±1.1	3.7±1.0	3.8±1.1	3.9±1.0	4.0±1.0	3.3±0.9	2.8±0.7
**FEV_1_ (L)**	2.8±0.8	2.7±0.8	2.8±0.9	3.0±0.8	2.2±0.6	2.4±0.7	2.4±0.7	3.0±0.9	2.7±0.8	3.0±0.9	3.0±0.9	3.1±0.9	2.5±0.8	2.3±0.6

**TABLE 1:** Characteristics of participants from 41 sites of the BOLD Study with acceptable and repeatable post-bronchodilator spirometry and occupational exposure data (continued)

**BOLD site**	**India (Mysore)**	**India (Pune)**	**Jamaica**	**Kyrgyzstan (Chui)**	**Kyrgyzstan (Naryn)**	**Malawi (Blantyre)**	**Malawi (Chikwawa)**	**Malaysia (Penang)**	**Morocco (Fes)**	**Netherlands (Maastricht)**	**Nigeria (Ife)**	**Norway (Bergen)**	**Pakistan (Karachi)**	**Philippines (Manila)**
**Subjects, n**	604	845	578	891	859	403	448	663	768	590	884	658	610	892
**Age (year)**	46.7±7.3	52.4±9.9	55.9±11.6	52.4±9.1	52.7±10.2	52.2±10.0	53.7±10.5	54.5±9.5	55.1±10.3	57.5±10.7	55.3±12.0	59.8±12.6	51.6±9.6	52.4±10.2
**Height (cm)**	158.6±6.6	158.8±8.9	165.7±8.8	161.1±8.8	160.1±8.7	161.2±8.2	161.6±9.1	158.8±8.2	161.7±9.1	169.9±9.6	162.7±7.7	170.9±9.5	159.5±9.6	156.4±8.6
**BMI (kg·m^−2^)**	24.7±3.8	22.1±3.8	27.5±6.6	28.4±5.7	27.0±5.0	25.0±5.4	21.8±3.9	26.1±4.5	27.9±5.3	27.4±4.5	25.3±5.4	26.5±4.3	26.5±5.5	24.9±4.7
**Sex (male)**	42.7	59.4	42	31.4	38.2	40	51.3	51.3	46.1	50.9	39.1	49.2	44.1	42.4
**Never-smokers**	89.7	87.5	62.3	70.4	75.4	86.3	69.7	74.5	72.1	32.9	88.6	35.7	74.1	46.6
**<20 pack-years**	8.4	11.7	19.4	14.5	14.9	12.7	27.8	12.4	13.4	34.8	10.4	38.6	13.3	35.4
**≥20 pack-years**	1.8	0.8	18.3	15.2	9.7	1	2.6	13.1	14.5	32.4	1	25.7	12.5	17.9
**Chronic cough**	1.7	1.9	4.2	10.2	10.7	2.2	1.4	4.5	9.8	5.3	0.5	7.9	11.4	4.5
**Chronic phlegm**	1.7	1.4	4.3	7	7.8	0.3	0.5	4.2	7.9	3.2	0.3	10	10.1	11.4
**Wheeze**	0.8	4.7	16.4	14.5	13.4	8	3	6.6	12.1	16.7	2.2	23.7	11.5	15.5
**Dyspnoea**	0	6.6	12.9	14.2	21.1	2	1.3	9.2	14.5	9.5	3.5	5.4	30.7	21.8
**FEV_1_/FVC (%)**	79.5±7.4	79.7±8.1	78.4±9.2	77.4±8.1	78.0±7.2	78.2±7.8	76.3±9.2	81.0±6.8	78.1±8.3	74.6±10.0	78.5±8.4	74.9±8.8	80.1±9.7	79.0±8.9
**FVC (L)**	2.6±0.7	2.7±0.7	2.9±0.8	3.4±0.9	3.5±0.9	3.0±0.7	3.1±0.7	2.7±0.7	3.3±0.9	4.0±1.1	2.7±0.7	3.9±1.1	2.5±0.8	2.6±0.7
**FEV_1_ (L)**	2.1±0.6	2.2±0.6	2.3±0.7	2.6±0.7	2.7±0.7	2.3±0.6	2.4±0.6	2.2±0.6	2.6±0.7	2.9±0.9	2.1±0.6	2.9±0.9	2.0±0.6	2.1±0.6

**TABLE 1:** Characteristics of participants from 41 sites of the BOLD Study with acceptable and repeatable post-bronchodilator spirometry and occupational exposure data (continued)

**BOLD site**	**Philippines (Nampicuan & Talugtug)**	**Poland (Krakow)**	**Portugal (Lisbon)**	**Saudi Arabia (Riyadh)**	**South Africa (Uitsig & Ravensmead)**	**Sri Lanka**	**Sudan (Gezira)**	**Sudan (Khartoum)**	**Sweden (Uppsala)**	**Trinidad & Tobago**	**Tunisia (Sousse)**	**Turkey (Adana)**	**USA (Lexington, KY)**
**Subjects, n**	722	526	711	700	846	1035	590	517	547	1097	661	806	508
**Age (year)**	54.1±10.5	55.7±11.5	63.3±11.3	50.3±7.7	54.2±10.5	53.7±9.5	53.7±10.2	54.0±10.4	58.4±10.9	54.1±10.8	53.0±9.1	53.6±10.4	56.6±9.9
**Height (cm)**	158.7±8.6	167.0±8.5	160.7±9.4	162.5±8.9	161.6±8.9	156.4±8.8	163.0±10.8	165.5±9.5	171.0±9.7	165.1±11.3	163.2±9.4	160.7±9.3	167.1±9.9
**BMI (kg·m^−2^)**	21.5±3.9	27.7±4.7	28.2±4.6	31.2±6.0	27.9±7.5	24.2±4.6	27.3±17.1	26.5±6.4	27.0±4.4	29.1±10.0	29.2±5.6	29.6±5.3	30.8±6.8
**Sex (male)**	49.3	50.6	46.6	53.6	37.2	44.9	51.5	59.4	51.7	39.8	46.8	48.3	40.6
**Never-smokers**	46.8	38.2	59.5	73.1	32.3	78.1	74.3	76	39.1	72.4	57.8	45.2	35.8
**<20 pack-years**	26.2	28.5	16.6	11.7	48.1	17.5	19.9	17	38.8	14.2	12.3	23.3	21.3
**≥20 pack-years**	27	33.3	23.9	15.1	19.6	4.4	5.8	7	22.1	13.4	30	31.5	42.9
**Chronic cough**	7.1	8.2	10.6	12.1	11.5	6.6	2.6	4.1	7.9	7.5	11.4	7.8	19.5
**Chronic phlegm**	9.6	7.8	13.1	12.9	13.7	10.9	3.8	4.6	11.5	3.7	15.4	8.7	16.3
**Wheeze**	28	26.3	27.9	40.7	27.7	30.2	19.9	8.5	25.4	11.8	25	35	44.1
**Dyspnoea**	25.5	23.7	14.6	22	29.2	26.8	8	6.7	5.1	8.7	16.4	23.3	20
**FEV_1_/FVC (%)**	77.0±10.6	75.1±9.2	75.8±9.0	82.6±6.0	75.6±11.1	79.7±8.7	80.1±7.2	77.9±8.4	76.3±8.0	79.6±7.6	80.0±7.5	75.8±8.7	76.2±9.4
**FVC (L)**	2.7±0.8	3.8±1.0	3.2±0.9	3.0±0.8	2.9±0.8	2.3±0.6	3.0±0.8	2.9±0.8	4.0±1.1	2.7±0.8	3.4±0.9	3.4±0.9	3.4±1.0
**FEV_1_ (L)**	2.1±0.7	2.9±0.9	2.4±0.8	2.5±0.7	2.2±0.7	1.9±0.5	2.4±0.7	2.3±0.6	3.0±0.9	2.2±0.7	2.7±0.8	2.6±0.7	2.6±0.9

Data are presented as mean±sd or the percentage, unless otherwise indicated. BOLD: Burden of Obstructive Lung Disease; BMI: body mass index; FEV_1_: forced expiratory volume in 1 s; FVC: forced vital capacity.

### Occupational exposure

Participants were asked if they had ever worked, for at least 3 months, in at least one of 11 work settings likely to be associated with significant exposures to particulates or fumes and loss of lung function. These were 1) farming; 2) flour, feed or grain milling; 3) cotton or jute processing; 4) hard-rock mining; 5) coal mining; 6) sandblasting; 7) working with asbestos; 8) chemical or plastics manufacturing; 9) foundry or steel milling; 10) welding; and 11) firefighting. In addition, they were asked about their longest-held job, which was coded using the International Standard Classification of Occupations (ISCO-88) (supplementary table S1) [6]. Based on expert opinion, these self-reported occupational data were used to group occupations into three categories according to likely exposure to organic dusts, inorganic dusts and fumes. For each occupation and category of exposure, we calculated the total number of cumulative years of exposure based on the self-reported number of years worked in each setting.

### Respiratory symptoms and lung function

Chronic cough was defined as a frequent cough, without having a cold, on most days for at least 3 months each year. Chronic phlegm was defined as a frequent production of phlegm, without a cold, on most days for at least 3 months each year. Wheeze was defined as having had any whistling in the chest at any time in the last 12 months. Dyspnoea was assessed using the modified Medical Research Council dyspnoea scale as breathlessness at least when walking more slowly than people of the same age or sufficient to have to stop walking [7].

Lung function testing was undertaken using an EasyOne spirometer (NDD Medizintechnik AG, Zurich, Switzerland) and each participant performed between three and eight manoeuvres. FEV_1_ and FVC were measured before and after the delivery of 200 μg of salbutamol through a metered-dose inhaler, *via* a spacer. All lung function measurements were individually evaluated at the coordinating centre. To be considered usable for analysis, the measurements had to fulfil the following criteria, based on the American Thoracic Society (ATS) criteria at the time the study began [8]: 1) no hesitation, *i.e.* back-extrapolated volume <150 mL and peak expiratory flow time <120 ms; 2) complete blow, *i.e.* lasting ≥6 s or evidence of clear plateau (end-of-time volume <40 mL; 3) no artefact affecting the FEV_1_ or FVC (*e.g.* cough, zero flow error); and 4) the two best blows within 200 mL of each other. We used FEV_1_/FVC as a marker of airflow obstruction, and FVC as a proxy for lung volumes.

### Statistical analysis

Participants with no exposure to any of the considered work settings were used as the reference group for all analyses. We used regression analysis to assess the association of respiratory symptoms (logistic) and lung function (linear) with occupation and occupational exposure category. All regression models were adjusted for sex, age (years) and smoking status (never-smoker, <20 pack-years and ≥20 pack-years). Models with FVC as the outcome were further adjusted for height (cm), and those with wheeze or dyspnoea as outcomes were also adjusted for body mass index [9]. Exposure-response trends were evaluated using both continuous and categorical exposure variables, with the median of years of exposure used as the cut-off value for examining cumulative exposures. The effect size for each association was estimated for each site, and the estimates from all sites were combined through random effects meta-analysis. The level of between-site heterogeneity was summarised by the I^2^ statistic [10].

In sensitivity analyses, we examined the associations of lung function with each of the three occupational categories among never-smokers only. In addition, we re-ran analyses by sex and by gross national income groups (HICs and LMICs). Stata 15 (Stata Corp., College Station, TX, USA) was used to perform all data analyses. Weights were used to account for sampling strategy. All results were considered statistically significant at p<0.05.

## Results

Table 1 summarises the characteristics of the study participants from the 41 BOLD sites included in this report. The mean age across sites ranged from 46.7 to 63.3 years; 47.4% of participants were male. The mean FEV_1_/FVC varied from 74.3% in Austria (Salzburg) to 82.6% in Saudi Arabia (Riyadh) and the mean FVC from 2.3 L in Sri Lanka to 4.0 L in Austria (Salzburg), Iceland (Reykjavik), the Netherlands (Maastricht) and Sweden (Uppsala). The highest proportion of people likely exposed to organic dusts in the workplace was in a rural site in India (Pune, 87.9%), while workers likely exposed to inorganic dust were more common in Poland (Krakow, 26.4%) and those likely exposed to fumes were in the USA (Lexington, KY; 27.6%). The proportion of participants who did not work in any occupation with exposure to dusts or fumes varied across sites from 8.8% in India (Pune) to 98.2% in India (Mumbai). Further details on the distributions among the 11 occupations in each site can be seen in table 2.

**TABLE 2:** Participants from 41 sites of the BOLD study across 11 work settings likely linked to significant exposure to particulates or fumes and loss of lung function

**BOLD site**	**Albania (Tirana)**	**Algeria (Annaba)**	**Australia (Sydney)**	**Austria (Salzburg)**	**Benin (Sèmè-Kpodji)**	**Cameroon (Limbe)**	**Canada (Vancouver)**	**China (Guangzhou)**	**England (London)**	**Estonia (Tartu)**	**Germany (Hannover)**	**Iceland (Reykjavik)**	**India (Kashmir)**	**India (Mumbai)**
**Subjects, n**	939	890	541	1253	698	331	827	461	675	613	680	757	760	439
**Unexposed to any high-risk occupation**	486 (51.8)	356 (40.0)	442 (81.7)	831 (66.3)	650 (93.1)	108 (32.6)	678 (82.0)	396 (85.9)	596 (88.3)	439 (71.6)	491 (72.2)	306 (40.4)	495 (65.1)	431 (98.2)
**Occupational exposure to organic dusts**	352 (37.5)	33 (3.7)	40 (7.4)	307 (24.5)	9 (1.3)	194 (58.6)	80 (9.7)	20 (4.3)	36 (5.3)	99 (16.2)	71 (10.4)	369 (48.8)	259 (34.1)	7 (1.6)
Farming	334 (35.6)	24 (2.7)	31 (5.7)	288 (23.0)	8 (1.2)	194 (58.6)	66 (8.0)	1 (0.2)	27 (4.0)	91 (14.9)	51 (7.5)	350 (46.2)	259 (34.1)	0 (0.0)
Flour, feed or grain milling	7 (0.8)	9 (1.0)	7 (1.3)	37 (3.0)	1 (0.1)	0 (0.0)	21 (2.5)	3 (0.7)	5 (0.7)	13 (2.1)	16 (2.4)	43 (5.7)	0 (0.0)	0 (0.0)
Cotton or jute processing	15 (1.6)	0 (0.0)	6 (1.1)	19 (1.5)	0 (0.0)	0 (0.0)	4 (0.5)	16 (3.5)	7 (1.0)	4 (0.7)	13 (1.9)	18 (2.4)	0 (0.0)	7 (1.6)
**Occupational exposure to inorganic dusts**	68 (7.2)	24 (2.7)	30 (5.6)	59 (4.7)	17 (2.4)	8 (2.4)	44 (5.3)	8 (1.7)	18 (2.7)	24 (3.9)	58 (8.5)	62 (8.2)	4 (0.5)	0 (0.0)
Hard-rock mining	20 (2.1)	3 (0.3)	4 (0.7)	16 (1.3)	3 (0.4)	2 (0.6)	14 (1.7)	3 (0.7)	3 (0.4)	5 (0.8)	6 (0.9)	27 (3.6)	0 (0.0)	0 (0.0)
Coal mining	16 (1.7)	1 (0.1)	3 (0.6)	6 (0.5)	3 (0.4)	0 (0.0)	2 (0.2)	2 (0.4)	1 (0.2)	1 (0.2)	11 (1.6)	1 (0.1)	0 (0.0)	0 (0.0)
Sandblasting	10 (1.1)	7 (0.8)	1 (0.2)	15 (1.2)	0 (0.0)	1 (0.3)	8 (1.0)	0 (0.0)	5 (0.7)	4 (0.7)	14 (2.1)	13 (1.7)	4 (0.5)	0 (0.0)
Working with asbestos	34 (3.6)	15 (1.7)	26 (4.8)	30 (2.4)	14 (2.0)	5 (1.5)	26 (3.1)	3 (0.7)	12 (1.8)	19 (3.1)	36 (5.3)	29 (3.8)	0 (0.0)	0 (0.0)
**Occupational exposure to fumes**	57 (6.1)	194 (21.8)	64 (11.8)	162 (12.9)	10 (1.4)	4 (1.2)	59 (7.3)	42 (9.1)	41 (6.1)	88 (14.4)	115 (16.9)	177 (23.4)	3 (0.4)	1 (0.2)
Chemical/ plastics manufacturing	22 (2.3)	14 (1.6)	28 (5.2)	44 (3.5)	0 (0.0)	0 (0.0)	25 (3.0)	11 (2.4)	24 (3.6)	45 (7.3)	50 (7.4)	78 (10.3)	1 (0.1)	0 (0.0)
Foundry or steel milling	11 (1.2)	156 (17.5)	11 (2.1)	22 (4.1)	0 (0.0)	0 (0.0)	20 (2.4)	18 (3.9)	8 (1.2)	17 (1.4)	16 (2.6)	30 (4.4)	1 (0.1)	0 (0.0)
Welding	24 (2.6)	50 (5.6)	28 (5.2)	77 (6.2)	10 (1.4)	4 (1.2)	19 (2.3)	16 (3.5)	14 (2.1)	34 (5.6)	62 (9.1)	95 (12.6)	0 (0.0)	1 (0.2)
Firefighting	3 (0.3)	7 (0.8)	6 (1.2)	52 (4.2)	0 (0.0)	0 (0.0)	6 (0.7)	1 (0.2)	2 (0.3)	10 (1.6)	8 (1.2)	11 (1.5)	1 (0.1)	0 (0.0)

**TABLE 2:** Participants from 41 sites of the BOLD Study across 11 work settings likely linked to significant exposure to particulates or fumes and loss of lung function (continued)

**BOLD site**	**India (Mysore)**	**India (Pune)**	**Jamaica**	**Kyrgyzstan (Chui)**	**Kyrgyzstan (Naryn)**	**Malawi (Blantyre)**	**Malawi (Chikwawa)**	**Malaysia (Penang)**	**Morocco (Fes)**	**Netherlands (Maastricht)**	**Nigeria (Ife)**	**Norway (Bergen)**	**Pakistan (Karachi)**	**Philippines (Manila)**
**Subjects, n**	604	845	578	891	859	403	448	663	768	590	884	658	610	892
**Unexposed to any high-risk occupation**	506 (83.8)	74 (8.8)	353 (61.1)	535 (60.0)	240 (27.9)	204 (50.6)	396 (88.4)	557 (84.0)	514 (66.9)	444 (75.3)	417 (47.2)	419 (63.7)	556 (91.2)	674 (75.6)
**Occupational exposure to organic dusts**	91 (15.1)	743 (87.9)	172 (29.8)	317 (35.6)	617 (71.8)	189 (46.9)	28 (6.3)	69 (10.4)	209 (27.2)	55 (9.3)	424 (49.1)	95 (14.4)	24 (3.9)	159 (17.8)
Farming	91 (15.1)	739 (87.5)	163 (28.2)	307 (34.5)	617 (71.8)	182 (45.2)	21 (4.7)	52 (7.8)	184 (24.0)	38 (6.4)	416 (47.1)	64 (9.7)	21 (3.44)	128 (14.4)
Flour, feed or grain milling	0 (0.0)	12 (1.4)	1 (0.2)	8 (0.9)	1 (0.1)	4 (1.0)	0 (0.0)	3 (0.5)	17 (2.2)	14 (2.4)	24 (2.7)	21 (3.2)	0 (0.0)	11 (1.2)
Cotton or jute processing	98 (16.2)	5 (0.6)	8 (1.4)	17 (1.9)	0 (0.0)	9 (2.2)	8 (1.8)	16 (2.4)	18 (2.3)	7 (1.2)	16 (1.8)	24 (3.7)	3 (0.5)	29 (3.3)
**Occupational exposure to inorganic dusts**	1 (0.2)	60 (7.1)	26 (4.5)	24 (2.7)	0 (0.0)	13 (3.2)	14 (3.1)	9 (1.4)	19 (2.5)	49 (8.3)	46 (5.2)	87 (13.2)	10 (1.6)	13 (1.5)
Hard-rock mining	0 (0.0)	48 (5.7)	0 (0.0)	6 (0.7)	0 (0.0)	7 (1.7)	7 (1.6)	4 (0.6)	8 (1.0)	4 (0.7)	15 (1.7)	9 (1.4)	0 (0.0)	4 (0.5)
Coal mining	0 (0.0)	2 (0.2)	0 (0.0)	14 (1.6)	0 (0.0)	2 (0.5)	1 (0.2)	3 (0.5)	2 (0.3)	7 (1.2)	3 (0.3)	4 (0.6)	0 (0.0)	0 (0.0)
Sandblasting	0 (0.0)	7 (0.8)	1 (0.2)	3 (0.3)	0 (0.0)	0 (0.0)	0 (0.0)	4 (0.6)	3 (0.4)	2 (0.3)	12 (1.4)	20 (3.0)	0 (0.0)	5 (0.6)
Working with asbestos	1 (0.2)	5 (0.6)	25 (4.3)	2 (0.2)	0 (0.0)	6 (1.5)	6 (1.3)	1 (0.2)	10 (1.3)	39 (6.6)	24 (2.7)	73 (11.1)	10 (1.6)	6 (0.7)
**Occupational exposure to fumes**	3 (0.5)	61 (7.2)	44 (7.6)	42 (4.7)	3 (0.4)	10 (2.5)	5 (1.1)	33 (5.0)	48 (6.3)	84 (14.2)	36 (4.1)	156 (23.7)	13 (2.1)	59 (6.6)
Chemical/ plastics manufacturing	0 (0.0)	18 (2.1)	13 (2.3)	18 (2.0)	0 (0.0)	2 (0.5)	1 (0.2)	20 (3.0)	15 (2.0)	43 (7.3)	8 (0.9)	102 (15.5)	4 (0.7)	19 (2.1)
Foundry or steel milling	2 (0.3)	43 (5.1)	0 (0.0)	13 (1.5)	0 (0.0)	1 (0.3)	1 (0.2)	8 (1.2)	12 (1.6)	92 (12.2)	13 (1.5)	26 (4.4)	2 (0.3)	6 (0.7)
Welding	1 (0.2)	4 (0.5)	29 (5.0)	12 (1.4)	3 (0.4)	3 (0.7)	3 (0.7)	7 (1.1)	25 (3.3)	35 (5.9)	22 (2.5)	64 (9.7)	8 (1.3)	33 (3.7)
Firefighting	0 (0.0)	0 (0.0)	3 (0.5)	0 (0.0)	0 (0.0)	4 (1.0)	0 (0.0)	0 (0.0)	0 (0.0)	4 (0.7)	2 (0.2)	6 (0.9)	0 (0.0)	6 (0.7)

**TABLE 2:** Participants from 41 sites of the BOLD Study across 11 work settings likely linked to significant exposure to particulates or fumes and loss of lung function (continued)

**BOLD site**	**Philippines (Nampicuan & Talugtug)**	**Poland (Krakow)**	**Portugal (Lisbon)**	**Saudi Arabia (Riyadh)**	**South Africa (Uitsig & Ravensmead)**	**Sri Lanka**	**Sudan (Gezira)**	**Sudan (Khartoum)**	**Sweden (Uppsala)**	**Trinidad & Tobago**	**Tunisia (Sousse)**	**Turkey (Adana)**	**USA (Lexington, KY)**
**Subjects, n**	722	526	711	700	846	1035	590	517	547	1097	661	806	508
**Unexposed to any high-risk occupation**	145 (20.1)	193 (36.7)	544 (76.5)	617 (88.1)	641 (75.8)	715 (69.1)	265 (44.9)	322 (62.3)	381 (69.7)	909 (82.9)	613 (92.7)	339 (42.1)	206 (40.6)
**Occupational exposure to organic dusts**	574 (79.5)	176 (33.5)	132 (18.6)	60 (8.6)	92 (10.9)	228 (22.0)	291 (49.3)	124 (24.0)	91 (16.6)	96 (8.8)	23 (3.5)	436 (54.1)	211 (41.5)
Farming	559 (77.4)	164 (31.2)	120 (16.9)	60 (8.6)	21 (2.5)	218 (21.1)	284 (48.1)	116 (22.4)	86 (15.7)	92 (8.4)	13 (2.0)	394 (48.9)	204 (40.2)
Flour, feed or grain milling	8 (1.1)	18 (3.4)	2 (0.3)	0 (0.0)	16 (1.9)	3 (0.3)	13 (2.2)	8 (1.2)	25 (4.6)	4 (0.4)	2 (0.3)	15 (1.9)	19 (3.7)
Cotton or jute processing	34 (4.7)	3 (0.6)	12 (1.7)	0 (0.0)	62 (7.3)	10 (1.0)	14 (2.4)	14 (2.7)	5 (1.0)	1 (0.1)	8 (1.2)	73 (9.1)	11 (2.2)
**Occupational exposure to inorganic dusts**	17 (2.4)	139 (26.4)	10 (1.4)	3 (0.4)	44 (5.2)	21 (2.0)	31 (5.3)	11 (2.1)	49 (9.0)	27 (2.5)	6 (0.9)	18 (2.2)	116 (22.8)
Hard-rock mining	4 (0.6)	27 (5.1)	2 (0.3)	0 (0.0)	8 (1.0)	9 (0.9)	26 (4.4)	4 (0.8)	8 (1.5)	2 (0.2)	5 (0.8)	7 (0.9)	15 (3.0)
Coal mining	14 (1.9)	117 (22.2)	2 (0.3)	0 (0.0)	3 (0.4)	3 (0.3)	5 (0.9)	0 (0.0)	1 (0.2)	0 (0.0)	1 (0.2)	4 (0.5)	78 (15.4)
Sandblasting	0 (0.0)	6 (1.1)	1 (0.1)	3 (0.4)	14 (1.7)	1 (0.1)	0 (0.0)	0 (0.0)	11 (2.0)	6 (0.6)	1 (0.2)	9 (1.1)	19 (3.7)
Working with asbestos	1 (0.1)	13 (2.5)	5 (0.7)	0 (0.0)	30 (3.6)	10 (1.0)	2 (0.3)	7 (1.4)	36 (6.6)	21 (1.9)	0 (0.0)	0 (0.0)	41 (8.1)
**Occupational exposure to fumes**	23 (3.2)	103 (19.6)	38 (5.3)	24 (3.4)	102 (12.0)	17 (1.6)	35 (5.9)	26 (5.0)	87 (15.9)	74 (6.8)	19 (2.9)	50 (6.2)	140 (27.6)
Chemical/plastics manufacturing	7 (1.0)	29 (5.5)	35 (3.5)	8 (1.1)	54 (6.4)	5 (0.5)	12 (2.0)	11 (2.1)	37 (6.8)	20 (1.8)	4 (0.6)	15 (1.9)	60 (11.8)
Foundry or steel milling	4 (0.6)	19 (3.6)	59 (9.0)	0 (0.0)	16 (1.9)	0 (0.0)	2 (0.3)	2 (0.4)	21 (3.8)	5 (0.5)	2 (0.3)	16 (2.0)	33 (6.5)
Welding	13 (1.8)	46 (8.8)	9 (1.3)	14 (2.0)	47 (5.6)	12 (1.2)	23 (3.9)	14 (2.7)	42 (7.7)	52 (4.7)	14 (2.1)	28 (3.5)	71 (14.0)
Firefighting	0 (0.0)	20 (3.8)	2 0.3)	2 (0.3)	1 (0.1)	0 (0.0)	2 (0.3)	3 (0.6)	9 (1.7)	2 (0.2)	1 (0.2)	4 (0.5)	24 (4.7)

Data presented as n (%), unless otherwise indicated. BOLD: Burden of Obstructive Lung Disease.

### Respiratory symptoms and occupational factors

**FIGURE 1:**
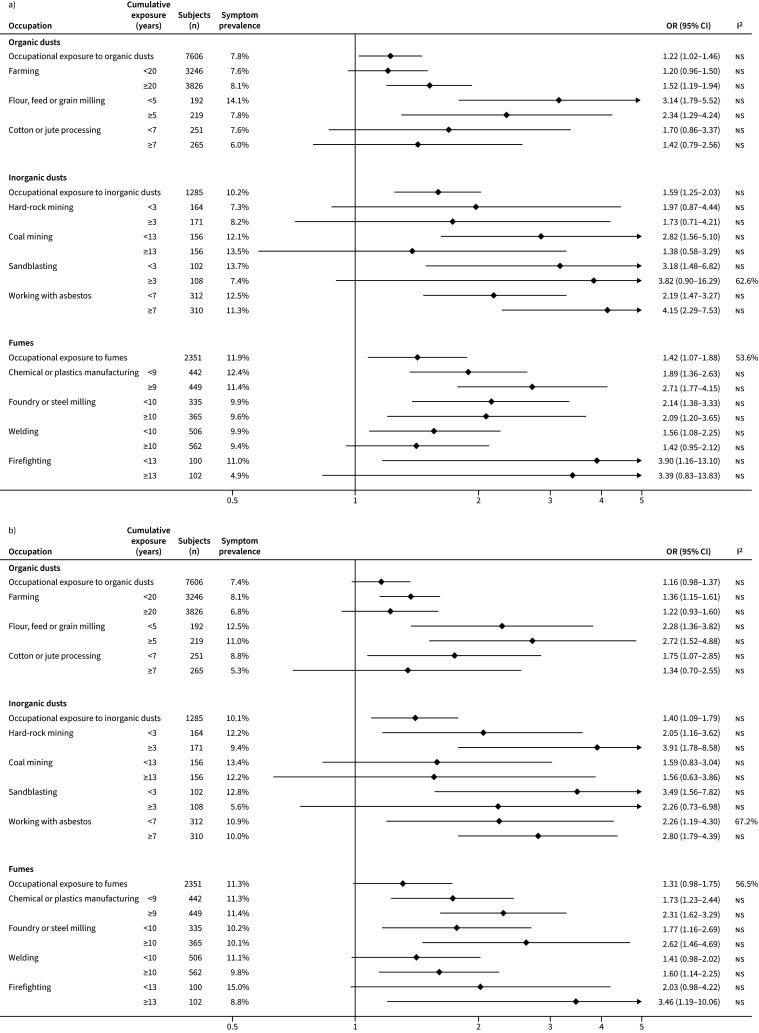
Association of a) chronic cough and b) chronic phlegm with high-risk occupations. ns: nonsignificant.

**FIGURE 2:**
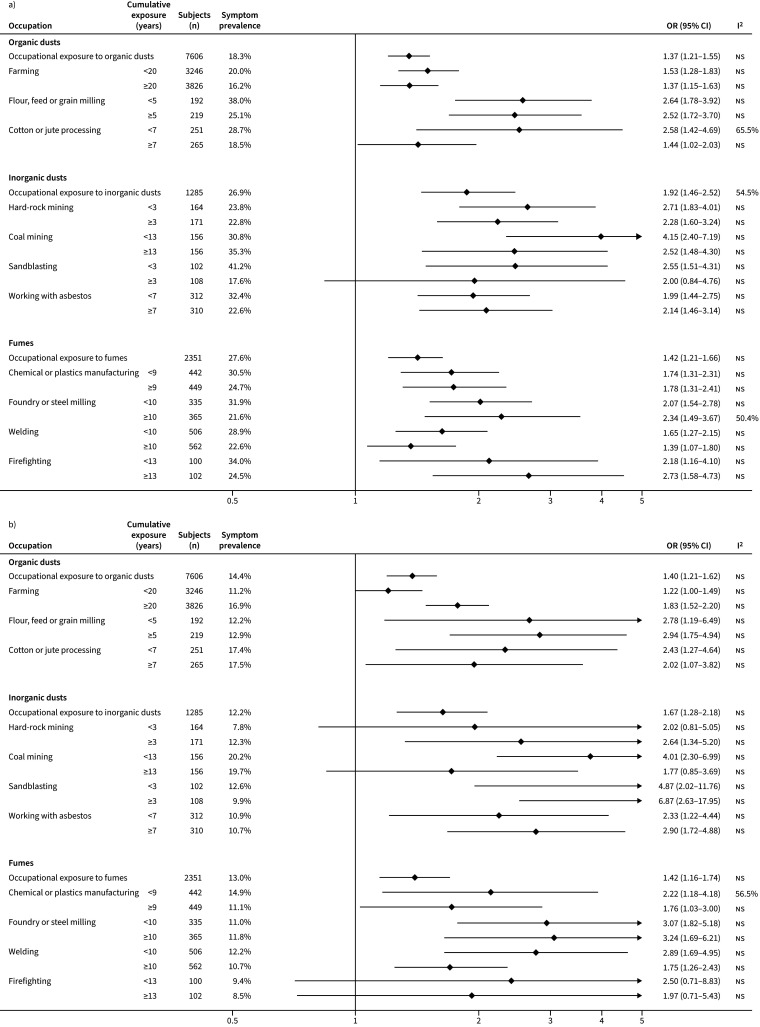
Association of a) wheeze and b) dyspnoea with high-risk occupations. ns: nonsignificant.

Figures 1 and 2 show the relationships between respiratory symptoms and occupational exposures. Overall, chronic cough, chronic phlegm, wheeze and dyspnoea were associated with most high-risk occupations. Farming, the most common occupation among participants, was associated with chronic cough (≥20 years: OR 1.52, 95% CI 1.19–1.94), chronic phlegm (<20 years: OR 1.36, 95% CI 1.15–1.61), wheeze (<20 years: OR 1.53, 95% CI 1.29–1.83; ≥20 years: OR 1.37, 95% CI 1.16–1.63) and dyspnoea (≥20 years: OR 1.83, 95% CI 1.53–2.20). Flour, feed or grain milling regardless of duration of exposure was associated with all respiratory symptoms studied. Working with asbestos for ≥7 years was clearly associated with chronic cough (OR 4.15, 95% CI 2.29–7.53). Hard-rock mining for ≥3 years was associated with chronic phlegm (OR 3.91, 95% CI 1.79–8.58). Coal mining was associated with wheeze (<13 years: OR 4.15, 95% CI 2.40–7.19). Sandblasting was highly associated with dyspnoea (<3 years: OR 4.87, 95% CI 2.02–11.76; ≥3 years: OR 6.87, 95% CI 2.63–17.95).

### Lung function and occupational factors

We found no significant associations between post-bronchodilator FEV_1_/FVC ratio or FVC and work in any of the high-risk occupations. FEV_1_/FVC was low in coal miners, sandblasters, chemical or plastic processors and steel millers with long durations of exposure, but these associations were not statistically significant. Moreover, there was no evidence of exposure-response associations of either post-bronchodilator lung function measures with any of the specific occupations (supplementary table S2).

Sensitivity analyses of lung function among men in HICs indicated that working in a job with organic dust exposure for ≥20 years was associated with a significantly decreased FEV_1_/FVC (β= −0.34%, 95% CI −0.42– −0.27%; supplementary table S3) and FVC (β= −0.18 L, 95% CI −0.32– −0.04 L; supplementary table S4). In LMICs, we found that men working in an organic dust job for a longer duration had a lower FEV_1_/FVC than in HICs; however, this finding was not statistically significant (β= −1.01%, 95% CI −2.77–0.75%) and there was high heterogeneity across the LMIC sites (I^2^=92.2%, p<0.001). There were no significant associations, in sensitivity analyses, between work with inorganic dusts and FEV_1_/FVC (supplementary table S5). Among never-smoking women in HICs, workplace exposure to inorganic dusts for ≥6 years was associated with greater FVC (β=0.60 L, 95% CI 0.53–0.66 L; supplementary table S6). Among men exposed to fumes at work for ≥11 years in LMICs, a sensitivity analysis showed a significant association with a lower FEV_1_/FVC (β= −0.29%, 95% CI −0.39– −0.17%; supplementary table S7). In contrast, there was no such association in HICs. There was no significant association between workplace exposure to fumes with FVC (supplementary table S8).

## Discussion

In this large international population-based study we found that respiratory symptoms were associated with working in settings where exposure to dusts and fumes is likely to be high. Overall, these findings agree with two recent reviews reporting significant relationships of chronic bronchitis and breathlessness with occupational exposures to organic dusts, inorganic (mineral) dusts or fumes [1, 2]. In contrast, we found no consistent associations between occupational exposures and measures of lung function. Lung function was lower in miners and chemical or plastic processors with long durations of exposure, but these differences were not statistically significant. One explanation for the association of chronic respiratory symptoms without significant lung function differences may be irritation of the airways leading to chronic bronchitis without obstruction. These findings are similar to those we found previously in the BOLD study, where chronic phlegm but not chronic airflow obstruction was more likely to occur among users than among never-users of solid fuels [11]. The “English Hypothesis” of a strong link between bronchitis and obstruction was largely discredited by the study of Fletcher and Peto [12], and the lack of a strong association in this study should not be entirely unexpected. Another explanation could be the occurrence of occupational asthma, presenting with wheeze and breathlessness without affecting post-bronchodilator lung function. This can be induced by substances in workplaces such as animal dusts, flour, chemicals and metals [13]. In addition, non-differential misclassification of exposure might have hampered the ability to detect a statistically significant association.

Stratifying analyses by sex, gross national income and smoking status among male participants in HICs showed that working in an organic dust job for ≥20 years was associated with slightly lower FEV_1_/FVC and FVC. In a combined analysis of the European Community Respiratory Health Survey (ECRHS) and the Swiss Cohort Study on Air Pollution and Lung and Heart Diseases in Adults (SAPALDIA), a decline in FEV_1_/FVC was associated with exposure to organic dust [14]. However, this association was not evident among never-smokers, suggesting that this relationship may have been due to residual confounding by smoking. A population-based study in Denmark reported an increased prevalence of COPD, defined by the lower limit normal (LLN) of FEV_1_/FVC, among workers exposed to high levels (≥15 years) of organic dust [15]. In LMICs, we found that men working in an organic dust job for ≥20 years had a greater, but insignificant, decrement in FEV_1_/FVC. However, this finding was highly heterogeneous across the LMIC sites. A potential explanation for these results is that farming was the most prevalent industry sector involving exposures to organic dusts in both HICs and LMICs. While HICs have similar commercial agriculture systems, LMICs are characterised by diverse and less intense agricultural practices, which might cause the significant heterogeneity in our LMIC analyses [16].

No significant association was observed in overall or sensitivity analyses between each lung function measure and inorganic dust exposure among men. Adjustment for passive smoking and education made no material difference to our findings. In the recent ECRHS and SAPALDIA report, FEV_1_/FVC decline was associated with mineral dust but again only if ever-smokers were included in the analysis [14]. The ECRHS had already reported no significant association of incident COPD, defined as FEV_1_/FVC<LLN, with inorganic (mineral) dust exposures [17]. For FVC, the only significant association we found was among never-smoking women exposed to inorganic dust for ≥6 years in HICs, whose FVC was on average 0.6 L greater than their unexposed counterparts. However, the women exposed in HICs were few (n=5) and diverse: two from the USA had worked with asbestos for 19 years and in hard-rock mining for 8 years, respectively; one from Estonia had worked with asbestos for 24 years; one from Germany had worked in sandblasting for 11 years; and one from Norway had worked with asbestos for 10 years. Therefore, the greater FVC in this group might have occurred by chance or might reflect a healthy worker effect [18].

The ECRHS study, which was conducted in 12 high-income European countries, reported an increased risk of COPD based on the LLN of FEV_1_/FVC among workers with occupational gas and fume exposures [17]. However, the association between FEV_1_/FVC decline and gases and fumes was not significant in the combined analysis of ECRHS and SAPALDIA [14]. In the current analysis of the BOLD study, we found no association between fumes and FEV_1_/FVC among men exposed to fumes for ≥11 years in HICs. This result is consistent with the recent findings of the UK Biobank, a large population-based study on lifetime job histories and spirometry-defined COPD that found no increased risk for fume-related jobs including chemical processing, metal processing and firefighting [19]. In contrast, there was a significant small effect on FEV_1_/FVC (decreased by 0.29%) among men in LMICs. An explanation for our study's contradictory findings might be related to different standards of industrial control between HICs and LMICs, where working conditions remain poor [20].

This study has several strengths. To the best of our knowledge, it is the first large population-based study covering both HICs and LMICs. We used a rigorous standardised protocol for data collection and lung function testing across all 41 sites. This is an advantage over published meta-analyses, which pooled findings from a mixture of study designs (cross-sectional surveys, case–control, longitudinal) with varying outcome definitions (either measured or based on self-report), because it reduces the heterogeneity across sites [1, 2]. Data collection was undertaken by certified technicians and trained interviewers. We tried to control for a potential recall bias by asking participants about their jobs (coded using the standard ISCO-88 classification) instead of their exposures at work. Furthermore, we undertook post-bronchodilator spirometry with centralised quality control for precision of spirometric measurements. In BOLD, about 96% of the manoeuvres met the ATS/European Respiratory Society 2005 goals for acceptability and 90% for repeatability [21].

We also recognise limitations. This study is cross-sectional, which makes it difficult to infer temporality and to distinguish causal relationships. Self-reported respiratory symptoms may be influenced by recall bias. Measurement error might have occurred due to misclassification of occupational histories and poor precision on durations of exposure, particularly in LMIC sites. For example, participants who worked on subsistence farms might not consider and report themselves as farmers. The inclusion of participants with an exposure ≥3 months but <1 year in the least exposed category may partly explain the lack of association among this category. However, this group of participants was relatively small (2% of those exposed to organic dust, 15% of those exposed to inorganic dust and 6% of those exposed to fumes) and the contrast between the highest exposure category and the non-exposed category should have been enough to detect a true association, if one existed. In addition, the questionnaire did not collect information on the intensity of each occupational exposure, which might limit analyses of the exposure-response relationship. Although the overall prevalence of occupational exposure to organic dust, inorganic dust and fumes was like that of other studies (37% *versus* 36–42%) [14], we are aware that the prevalence of exposure to inorganic dust is much lower than reported in those same studies. Regarding sensitivity analyses of lung function, we restricted these to just three main groups of dust and fume exposures rather than the 11 specified occupations because for some of these there were too few participants, particularly among women. Moreover, it is also noted that workers in industrial workplaces are generally exposed to combinations of respiratory hazards, which affects grouping of dusty and fume jobs so that we were not able to adjust our models for co-exposure to multiple occupational exposures. We are also conscious that our analysis is based on lung function measured using spirometry, not lung diffusion capacity or blood gases, which have been previously linked to occupational exposure to pesticides [22]. Finally, although the study is large, it does not necessarily imply representativeness of the population in each country.

We suggest a further longitudinal study on the association of occupational exposure with respiratory outcomes, which would more easily distinguish causal from non-causal relationships. In addition, to evaluate high-quality occupational exposure assessment, comprehensive data collection on exposure magnitude (*e.g.* dose, frequency and intensity) is suggested. Therefore, personal monitoring for a larger global prospective cohort and application of a job-exposure matrix are recommended [23, 24]. We also found clear evidence that occupational dusty jobs were related to chronic respiratory symptoms with, in some cases, effects on lung function. Further laboratory studies to understand the mechanism of how workplace exposures to dusts and fumes affect lung function are also suggested.

In conclusion, we found that exposure to selected work settings, which are thought to be associated with substantial exposures to particulates or fumes and loss of lung function, may increase the risk of chronic respiratory symptoms, without significant changes in spirometric measures of lung function. This does not mean that unlimited occupational exposure is acceptable or cannot have an effect on the lungs. It just suggests that, in this study, occupational exposures do not appear to be major determinants of low spirometric values, compared with other exposures. Because we are aware that many work settings were not included in the BOLD study and individual risk might be higher in certain settings, interventions to avoid or reduce occupational exposures are advised. Industrial hygiene is still important and respiratory surveillance should be encouraged among high-risk dusty and fume job workers, especially those living in LMICs.

## Supplementary material

10.1183/13993003.00469-2022.Supp1 **Please note:** supplementary material is not edited by the Editorial Office, and is uploaded as it has been supplied by the author.
Supplementary material ERJ-00469-2022.Supplement

## Shareable PDF

10.1183/13993003.00469-2022.Shareable1 This one-page PDF can be shared freely online.
Shareable PDF ERJ-00469-2022.Shareable
