# Supplementary material for: Association of respiratory symptoms and lung function with occupation in the multinational Burden of Obstructive Lung Disease (BOLD) study
Source: Eur Respir J. 2023 Jan 12;61(1):2200469. doi: 10.1183/13993003.00469-2022 (PMC9834632; doi:10.1183/13993003.00469-2022)
Supplement: Supplementary file 1 [file ERJ-00469-2022.Supplement.pdf]

Table S1. Categorisation of occupations collected by the BOLD Study.

High-risk occupations*	Longest held occupations (ISCO-88)**	
**Organic dust**		
Farming	6111	Field crop & vegetable growers
	6112	Gardeners, horticultural & nursery growers
	6121	Dairy & livestock producers
	6122	Poultry producers
	6129	Animal producers & related workers
	6130	Crop & animal producers
	6141	Forestry workers & logger
	6142	Charcoal burners & related workers
	8331	Motorised farm & forestry plant operators
	9211	Farm hands & labourers
	9212	Forestry labourers
Flour, feed or grain milling	8273	Grain & spice milling machine operators
	8274	Baked goods & cereal products machine operators
Cotton or jute processing	8261	Fibre preparing, spinning & winding machine operators
	8262	Weaving and knitting machine operators
	8263	Sewing machine operators
	8264	Bleaching, dyeing & cleaning machine operators
	8265	Fur & leather preparing machine operators†
	8269	Textile, fur & leather products machine operators†
**Inorganic dust**		
Hardrock mining	7111	Miners & quarry workers
	7113	Stone splitters, cutters & carvers
	9311	Mining & quarrying labourers
Coal mining	8111	Mining plant operators
	8112	Mineral ore & stone processing plant operators
	9311	Mining & quarrying labourers
Sandblasting	7143	Building structure cleaners
Working with asbestos	7124	Carpenters & joiners
	7131	Roofers
	7136	Plumbers & pipe fitters
	7137	Building & related electricians

Table S1. Categorisation of occupations collected by the BOLD Study (continued).

High-risk occupations*	Longest held occupations (ISCO-88)**	
**Fumes**		
Chemical or plastics manufacturing	8151	Crushing, grinding & chemical mixing machinery operators
	8152	Chemical heat-treating plant operators
	8153	Chemical filtering & separating equipment operators
	8154	Chemical still & reactor operators
	8155	Petroleum & natural gas refining plant operators
	8159	Chemical processing plant operators
	8221	Pharmaceutical & toiletry products machine operators
	8223	Metal finishing, plating & coating machine operators
	8224	Photographic products machine operators
	8229	Chemical products machine operators
	8231	Rubber products machine operators
	8232	Plastic products machine operators
	8284	Metal, rubber & plastic products assemblers
Foundry or steel milling	7211	Metal moulders & coremakers
	7221	Blacksmiths, hammer smiths & forging press workers
	8121	Ore & metal furnace operators
	8122	Metal melters, casters & rolling mill operators
	8123	Metal heat-treating plant operators
	8124	Metal drawers & extruders
Welding	7211	Metal moulders & coremakers
	7212	Welders & flame cutters
	7213	Sheetmetal workers
	7214	Structural metal preparers & erectors
	7215	Riggers & cable splicers
	7231	Motor vehicle mechanics & fitters
	7232	Aircraft engine mechanics & fitters
	7233	Agricultural or industrial machinery mechanics & fitters
Firefighting	5161	Firefighters

*High-risk occupations classified by US Department of Labour Dictionary of Occupational Titles (4th Ed., Rev. 1991)
**Longest held jobs classified by the International Standard Classification of Occupations (ISCO-88)
†This study included only participants responding to these codes and specifying work in cotton or jute processing industries

Table S2. Association of post-bronchodilator spirometric parameters with occupational variables.

		n	FEV_1/FVC (%)† Mean (SE)	β (95% CI)	I^2(%)	p-trend	FVC (%)†† Mean (SE)	β (95% CI)	I^2(%)	p-trend
Unexposed to any high-risk occupation		18,484	79.28 (0.19)	reference			3.09 (0.02)	reference		
Occupational exposure to organic dusts		7,612	77.32 (0.32)	-0.16 (-0.44 to 0.13)	NS		3.37 (0.03)	0.01 (-0.01 to 0.03)	NS	
Farming	<20 years	3,250	77.80 (0.51)	0.14 (-0.24 to 0.52)	NS	0.95	3.47 (0.03)	0.04 (-0.01 to 0.09)	77.9%	0.80
	≥20 years	3,828	76.37 (0.39)	0.04 (-0.49 to 0.58)	50.0%		3.25 (0.04)	0.02 (-0.02 to 0.06)	53.2%	
Flour, feed or grain milling	<5 years	192	77.27 (2.10)	-0.84 (-3.96 to 2.29	97.0%	0.84	3.92 (0.22)	-0.01 (-0.22 to 0.21)	96.1%	0.93
	≥5 years	219	74.64 (1.99)	-0.27 (-2.07 to 1.53)	93.3%		3.70 (0.22)	0.02 (-0.06 to 0.11)	86.9%	
Cotton or jute processing	<7 years	251	80.19 (0.90)	1.02 (-0.03 to 2.07)	66.7%	0.82	3.44 (0.31)	-0.12 (-0.24 to 0.00)	88.7%	0.88
	≥7 years	265	76.57 (0.97)	0.32 (-0.90 to 1.54)	87.1%		3.02 (0.08)	0.02 (-0.08 to 0.11)	85.3%	
Occupational exposure to inorganic dusts		1,287	76.58 (0.79)	-0.19 (-0.76 to 0.38)	NS		3.92 (0.08)	0.02 (-0.04 to 0.08)	64.1%	
Hard-rock mining	<3 years	164	74.58 (4.18)	-0.68 (-2.25 to 0.89)	78.4%	0.79	3.79 (0.09)	0.03 (-0.21 to 0.28)	96.9%	0.95
	≥3 years	171	71.61 (3.30)	-0.39 (-2.63 to 1.85)	94.1%		3.64 (0.22)	0.01 (-0.17 to 0.20)	94.0%	
Coal mining	<13 years	157	74.63 (0.53)	0.13 (-2.53 to 2.79)	97.0%	<0.05	3.79 (0.07)	-0.07 (-0.21 to 0.08)	90.7%	0.78
	≥13 years	156	74.05 (1.97)	-0.48 (-3.36 to 2.41)	94.3%		3.99 (0.12)	0.05 (-0.22 to 0.32)	97.1%	
Sandblasting	<3 years	102	73.20 (3.50)	0.15 (-3.39 to 3.69)	94.8%	0.27	3.86 (0.15)	-0.03 (-0.22 to 0.15)	92.7%	0.98
	≥3 years	108	70.83 (0.63)	-0.93 (-6.09 to 4.22)	99.4%		3.60 (0.05)	-0.03(-0.15 to 0.08)	89.9%	
Working with asbestos	<7 years	313	75.71 (0.65)	-1.80 (-4.60 to 1.01)	93.6%	0.72	4.17 (0.07)	-0.07 (-0.28 to 0.14)	95.0%	0.21
	≥7 years	310	78.10 (1.13)	0.19 (-1.25 to 1.63)	74.6%		3.96 (0.10)	-0.05 (-0.20 to 0.10)	90.8%	
Occupational exposure to fumes		2,352	76.42 (0.61)	0.13 (-0.65 to 0.92)	78.8%		3.78 (0.05)	0.00 (-0.03 to 0.04)	NS	
Chemical or plastics manufacturing	<9 years	443	76.29 (1.50)	0.13 (-0.84 to 1.10)	74.3%	0.28	3.68 (0.07)	0.01 (-0.06 to 0.08)	61.7%	0.86
	≥9 years	449	76.27 (1.31)	-0.65 (-1.84 to 0.54)	70.6%		3.59 (0.10)	-0.02 (-0.15 to 0.11)	89.7%	
Foundry or steel milling	<10 years	335	76.69 (0.96)	-0.68 (-2.14 to 0.79)	79.1%	0.16	3.90 (0.17)	0.04 (-0.10 to 0.18)	88.2%	0.06
	≥10 years	365	76.34 (2.68)	-1.70 (-4.73 to 1.33)	95.1%		3.84 (0.22)	-0.17* (-0.33 to -0.01)	87.9%	
Welding	<10 years	506	77.45 (0.71)	-0.25 (-1.79 to 1.28)	87.3%	0.07	4.03 (0.06)	0.04 (-0.27 to 0.35)	98.3%	0.32
	≥10 years	562	75.61 (1.59)	0.93 (-0.13 to 1.99)	76.4%		3.85 (0.07)	-0.04 (-0.12 to 0.04)	79.2%	
Firefighting	<13 years	100	77.20 (1.24)	1.73* (0.30 to 3.16)	86.8%	0.38	3.91 (0.10)	0.06 (-0.11 to 0.23)	94.0%	0.39
	≥13 years	102	76.38 (1.29)	-0.51 (-2.89 to 1.87)	96.9%		4.20 (0.11)	-0.05 (-0.18 to 0.08)	92.4%	

Means (SE) were from all 41-site participants together. †The coefficients (β) were adjusted for sex, age (years) and smoking status (never, <20 pack-years, ≥20 pack-years). ††The coefficients (β) were adjusted for sex, age (years), height (cm) and smoking status (never, <20 pack-years, ≥20 pack-years). *p<0.05; NS non-statistically significant (p≥0.05) heterogeneity (I^2); both p<0.05 and I^2=NS in bold.

Table S3. Association of FEV_1/FVC (%) with groups of organic dust jobs stratified by sex and sites' country economy.

		Men					Women				
		n	**Mean (SE)**	**β (95% CI)**	**I^2(%)**	**p-trend**	***n***	**Mean (SE)**	**β (95% CI)**	**I^2(%)**	**p-trend**
**Organic dusts (all)**											
Overall	unexposed to any	7,443	78.48 (0.28)	reference			11,041	79.99 (0.17)	reference		
	<20 years	2,005	77.48 (0.73)	0.13 (-0.46 to 0.71)	NS	0.65	1,626	78.90 (0.60)	0.07 (-0.40 to 0.55)	NS	0.80
	≥20 years	2,282	75.18 (0.45)	-0.95* (-1.48 to -0.42)	88.9%		1,699	78.53 (0.59)	0.75 (-0.26 to 1.77)	86.6%	
HICs	unexposed to any	3,023	78.15 (0.26)	reference			4,280	78.98 (0.17)	reference		
	<20 years	773	77.39 (0.77)	0.43 (-0.38 to 1.23)	NS	0.76	547	77.22 (0.44)	-0.29 (-0.96 to 0.38)	NS	0.83
	≥20 years	232	75.53 (0.87)	**-0.34* (-0.42** to **-0.27)**	**NS**		190	76.11 (1.19)	1.38 (-1.67 to 4.42)	90.1%	
LMICs	unexposed to any	4,420	78.56 (0.33)	reference			6,761	80.29 (0.21)	reference		
	<20 years	1,232	77.50 (0.86)	-0.05 (-0.83 to 0.74)	50.5%	0.56	1,079	79.45 (0.80)	0.23 (-0.40 to 0.85)	NS	0.85
	≥20 years	2,050	75.14 (0.48)	-1.01 (-2.77 to 0.75)	92.2%		1,509	78.96 (0.63)	0.41 (-0.57 to 1.39)	83.4%	
**Organic dusts (never-smokers)**											
Overall	unexposed to any	3,144	79.78 (0.27)	reference			8,437	80.27 (0.18)	reference		
	<20 years	819	76.81 (2.58)	0.16 (-0.65 to 0.97)	57.4%	0.53	1,227	79.34 (0.68)	0.07 (-0.39 to 0.54)	NS	0.50
	≥20 years	987	76.42 (0.60)	-0.36 (-1.37 to 0.65)	71.6%		1,471	79.20 (0.57)	0.44 (-0.63 to 1.51)	90.3%	
HICs	unexposed to any	1,126	79.94 (0.33)	reference			2,551	80.20 (0.16)	reference		
	<20 years	248	78.34 (0.65)	0.04 (-1.02 to 1.09)	NS	0.88	298	78.25 (0.50)	-0.35 (-1.13 to 0.43)	NS	<0.05
	≥20 years	82	79.02 (1.48)	-0.69 (-3.09 to 1.72)	80.8%		136	76.65 (1.48)	0.17 (-2.45 to 2.80)	89.0%	
LMICs	unexposed to any	2,018	79.75 (0.32)	reference			5,886	80.29 (0.21)	reference		
	<20 years	571	76.57 (2.95)	0.16 (-0.98 to 1.30)	65.3%	0.40	929	79.60 (0.85)	0.23 (-0.39 to 0.85)	NS	0.85
	≥20 years	905	76.24 (0.63)	-0.20 (-1.23 to 0.84)	62.6%		1,335	79.57 (0.59)	0.56 (-0.61 to 1.73)	90.7%	

HICs: high-income countries; LMICs low- and middle-income countries classified by the World Bank; never-smokers stratification included only participants reporting 'never-smoking'. All Means (SE) were from all 41-site participants; HIC Means (SE) were from 14 high-income site participants; LMIC Means (SE) were from 27 low- and middle-income site participants. The coefficients (β) were adjusted for age (years) and smoking status (never, <20 pack-years, ≥20 pack-years). *p<0.05; NS non-statistically significant (p≥0.05) heterogeneity (I^2); both p<0.05 and I^2=NS in bold.

Table S4. Association of FVC (L) with groups of organic dust jobs stratified by sex and sites' country economy

		Men					Women				
		n	**Mean (SE)**	**β (95% CI)**	**I^2(%)**	**p-trend**	***n***	**Mean (SE)**	**β (95% CI)**	**I^2(%)**	**p-trend**
**Organic dusts (all)**											
Overall	unexposed to any	7,443	3.60 (0.03)	reference			11,041	2.64 (0.04)	reference		
	<20 years	2,005	3.74 (0.07)	0.02 (-0.02 to 0.07)	NS	0.87	1,626	2.89 (0.05)	0.04 (0.00 to 0.08)	63.1%	<0.05
	≥20 years	2,282	3.57 (0.05)	-0.02 (-0.07 to 0.03)	52.0%		1,699	2.63 (0.05)	0.03 (-0.05 to 0.11)	88.3%	
HICs	unexposed to any	3,023	4.00 (0.04)	reference			4,280	2.84 (0.02)	reference		
	<20 years	773	4.06 (0.06)	0.01 (-0.05 to 0.06)	NS	<0.05	547	2.89 (0.05)	0.01 (-0.04 to 0.06)	NS	0.05
	≥20 years	232	3.73 (0.07)	**-0.18* (-0.32** to **-0.04)**	**NS**		190	2.72 (0.08)	0.02 (-0.17 to 0.21)	90.7%	
LMICs	unexposed to any	4,420	3.51 (0.05)	reference			6,761	2.58 (0.06)	reference		
	<20 years	1,232	3.67 (0.07)	0.03 (-0.04 to 0.09)	51.5%	0.14	1,079	2.89 (0.07)	0.05 (-0.01 to 0.11)	72.9%	0.15
	≥20 years	2,050	3.55 (0.05)	0.02 (-0.03 to 0.07)	NS		1,509	2.61 (0.06)	0.04 (-0.04 to 0.13)	86.6%	
**Organic dusts (never-smokers)**											
Overall	unexposed to any	3,144	3.53 (0.04)	reference			8,437	2.60 (0.04)	reference		
	<20 years	819	3.50 (0.20)	-0.06 (-0.16 to 0.03)	78.4%	0.69	1,227	2.87 (0.06)	0.04 (0.00 to 0.09)	62.4%	<0.05
	≥20 years	987	3.46 (0.05)	0.01 (-0.09 to 0.12)	81.5%		1,471	2.61 (0.04)	0.05 (-0.04 to 0.14)	92.0%	
HICs	unexposed to any	1,126	3.95 (0.07)	reference			2,551	2.69 (0.02)	reference		
	<20 years	248	4.00 (0.08)	0.00 (-0.10 to 0.10)	NS	<0.05	298	2.79 (0.06)	0.03 (-0.03 to 0.09)	NS	0.13
	≥20 years	82	3.66 (0.12)	-0.24* (-0.47 to -0.02)	67.2%		136	2.73 (0.10)	0.09 (-0.12 to 0.30)	91.4%	
LMICs	unexposed to any	2,018	3.45 (0.04)	reference			5,886	2.58 (0.06)	reference		
	<20 years	571	3.42 (0.22)	-0.08 (-0.21 to 0.05)	84.9%	0.59	929	2.89 (0.07)	0.05 (-0.01 to 0.12)	73.9%	0.17
	≥20 years	905	3.44 (0.06)	0.10 (-0.01 to 0.20)	80.5%		1,335	2.59 (0.05)	0.04 (-0.06 to 0.13)	92.5%	

HICs: high-income countries; LMICs low- and middle-income countries classified by the World Bank; never-smokers stratification included only participants reporting 'never-smoking'. All Means (SE) were from all 41-site participants; HIC Means (SE) were from 14 high-income site participants; LMIC Means (SE) were from 27 low- and middle-income site participants. The coefficients (β) were adjusted for age (years), height (cm) and smoking status (never, <20 pack-years, ≥20 pack-years). *p<0.05; NS non-statistically significant (p≥0.05) heterogeneity (I^2); both p<0.05 and I^2=NS in bold.

Table S5. Association of FEV_1/FVC (%) with groups of inorganic dust jobs stratified by sex and sites' country economy

		**Men**					**Women**				
		n	**Mean (SE)**	**β (95% CI)**	**I^2(%)**	**p-trend**	***n***	**Mean (SE)**	**β (95% CI)**	**I^2(%)**	**p-trend**
**Inorganic dusts (all)**											
Overall	unexposed to any	7,443	78.48 (0.28)	reference			11,041	79.99 (0.17)	reference		
	<6 years	550	76.48 (1.73)	0.19 (-0.91 to 1.30)	72.8%	0.73	77	75.10 (1.68)	0.09 (-2.43 to 2.61)	97.4%	0.56
	≥6 years	606	76.68 (1.26)	-0.04 (-0.78 to 0.71)	NS		54	77.50 (0.43)	0.73* (0.02 to 1.44)	98.4%	
HICs	unexposed to any	3,023	78.15 (0.26)	reference			4,280	78.98 (0.17)	reference		
	<6 years	344	75.47 (0.65)	0.54 (-1.12 to 2.21)	77.9%	0.13	43	74.85 (1.47)	-0.61 (-6.56 to 5.34)	97.3%	0.99
	≥6 years	235	75.75 (0.67)	0.91 (-0.33 to 2.14)	NS		14	77.59 (0.99)	-0.32 (-3.29 to 2.66)	96.1%	
LMICs	unexposed to any	4,420	78.56 (0.33)	reference			6,761	80.29 (0.21)	reference		
	<6 years	206	77.66 (3.63)	-0.15 (-1.72 to 1.42)	69.8%	0.08	34	75.96 (5.56)	0.82 (-0.92 to 2.57)	90.1%	0.42
	≥6 years	371	76.97 (1.66)	-0.59 (-1.31 to 0.13)	NS		40	77.42 (0.07)	1.53 (-1.13 to 4.20)	98.9%	
**Inorganic dusts (never-smokers)**											
Overall	unexposed to any	3,144	79.78 (0.27)	reference			8,437	80.27 (0.18)	reference		
	<6 years	154	79.96 (1.41)	1.12 (-0.48 to 2.72)	86.0%	0.11	43	76.82 (0.41)	-0.28 (-4.27 to 3.70)	99.4%	<0.01
	≥6 years	191	82.11 (1.38)	0.97 (-1.00 to 2.93)	93.8%		33	77.16 (0.60)	0.62 (-2.19 to 3.44)	98.3%	
HICs	unexposed to any	1,126	79.94 (0.33)	reference			2,551	80.20 (0.16)	reference		
	<6 years	83	77.57 (1.06)	0.63 (-1.92 to 3.18)	85.7%	0.79	18	78.23 (0.56)	0.86 (-4.94 to 6.66)	99.1%	0.25
	≥6 years	52	78.88 (0.80)	0.21 (-2.91 to 3.33)	94.3%		5	81.71 (2.26)	0.04 (-6.42 to 6.50)	98.5%	
LMICs	unexposed to any	2,018	79.75 (0.32)	reference			5,886	80.29 (0.21)	reference		
	<6 years	71	81.39 (1.73)	1.47 (-0.75 to 3.69)	87.1%	0.14	25	72.98 (0.14)	-1.44 (-6.94 to 4.05)	99.5%	<0.001
	≥6 years	139	82.69 (1.60)	1.42 (-1.33 to 4.17)	93.7%		28	75.61 (0.09)	0.84 (-2.41 to 4.09)	98.2%	

HICs: high-income countries; LMICs low- and middle-income countries classified by the World Bank; never-smokers stratification included only participants reporting 'never-smoking'. All Means (SE) were from all 41-site participants; HIC Means (SE) were from 14 high-income site participants; LMIC Means (SE) were from 27 low- and middle-income site participants. The coefficients (β) were adjusted for age (years) and smoking status (never, <20 pack-years, ≥20 pack-years). *p<0.05; NS non-statistically significant (p≥0.05) heterogeneity (I^2); both p<0.05 and I^2=NS in bold.

Table S6. Association of FVC (L) with groups of inorganic dust jobs stratified by sex and sites' country economy.

		Men					Women				
		n	**Mean (SE)**	**β (95% CI)**	**I^2(%)**	**p-trend**	***n***	**Mean (SE)**	**β (95% CI)**	**I^2(%)**	**p-trend**
**Inorganic dusts (all)**											
Overall	unexposed to any	7,443	3.60 (0.03)	reference			11,041	2.64 (0.04)	reference		
	<6 years	550	4.05 (0.05)	-0.02 (-0.14 to 0.10)	88.4%	0.18	77	2.87 (0.08)	0.04 (-0.19 to 0.27)	98.6%	0.09
	≥6 years	606	3.95 (0.11)	0.05 (-0.03 to 0.12)	66.4%		54	2.80 (0.08)	0.01 (-0.15 to 0.16)	95.7%	
HICs	unexposed to any	3,023	4.00 (0.04)	reference			4,280	2.84 (0.02)	reference		
	<6 years	344	4.38 (0.07)	-0.04 (-0.19 to 0.10)	80.5%	0.34	43	3.03 (0.06)	0.01 (-0.38 to 0.41)	98.3%	0.06
	≥6 years	235	4.18 (0.07)	0.05 (-0.06 to 0.15)	NS		14	3.17 (0.15)	0.23 (-0.11 to 0.57)	97.5%	
LMICs	unexposed to any	4,420	3.51 (0.05)	reference			6,761	2.58 (0.06)	reference		
	<6 years	206	3.65 (0.10)	-0.01 (-0.19 to 0.16)	91.1%	0.33	34	2.29 (0.26)	0.07 (-0.18 to 0.33)	98.2%	<0.001
	≥6 years	371	3.88 (0.14)	0.05 (-0.05 to 0.14)	73.5%		40	2.52 (0.09)	-0.13* (-0.26 to 0.00)	88.8%	
**Inorganic dusts (never-smokers)**											
Overall	unexposed to any	3,144	3.53 (0.04)	reference			8,437	2.60 (0.04)	reference		
	<6 years	154	3.90 (0.06)	-0.03 (-0.19 to 0.13)	90.4%	0.22	43	2.83 (0.01)	0.04 (-0.25 to 0.33)	99.2%	<0.05
	≥6 years	191	3.91 (0.17)	0.06 (-0.02 to 0.14)	71.3%		33	2.64 (0.02)	0.03 (-0.17 to 0.24)	96.6%	
HICs	unexposed to any	1,126	3.95 (0.07)	reference			2,551	2.69 (0.02)	reference		
	<6 years	83	4.49 (0.10)	0.01 (-0.20 to 0.21)	78.6%	<0.05	18	2.97 (0.02)	0.02 (-0.61 to 0.64)	99.4%	0.17
	≥6 years	52	4.11 (0.12)	0.11 (-0.01 to 0.23)	56.8%		5	3.50 (0.03)	**0.60* (0.53** to **0.66)**	**NS**	
LMICs	unexposed to any	2,018	3.45 (0.04)	reference			5,886	2.58 (0.06)	reference		
	<6 years	71	3.55 (0.15)	-0.05 (-0.29 to 0.18)	93.5%	0.75	25	2.45 (0.03)	0.08 (-0.21 to 0.38)	98.6%	<0.001
	≥6 years	139	3.87 (0.20)	0.03 (-0.07 to 0.13)	76.8%		28	2.35 (0.03)	-0.15* (-0.29 to 0.00)	87.4%	

HICs: high-income countries; LMICs low- and middle-income countries classified by the World Bank; never-smokers stratification included only participants reporting 'never-smoking'. All Means (SE) were from all 41-site participants; HIC Means (SE) were from 14 high-income site participants; LMIC Means (SE) were from 27 low- and middle-income site participants. The coefficients (β) were adjusted for age (years), height (cm) and smoking status (never, <20 pack-years, ≥20 pack-years). *p<0.05; NS non-statistically significant (p≥0.05) heterogeneity (I^2); both p<0.05 and I^2=NS in bold.

Table S7. Association of FEV_1/FVC (%) with groups of fume jobs stratified by sex and sites' country economy.

		Men					Women				
		n	**Mean (SE)**	**β (95% CI)**	**I^2(%)**	**p-trend**	***n***	**Mean (SE)**	**β (95% CI)**	**I^2(%)**	**p-trend**
**Fumes (all)**											
Overall	unexposed to any	7,443	78.48 (0.28)	reference			11,041	79.99 (0.17)	reference		
	<11 years	951	77.62 (0.69)	0.22 (-0.74 to 1.18)	74.4%	0.45	229	77.57 (1.10)	-0.51 (-1.63 to 0.60)	82.4%	<0.05
	≥11 years	1,002	75.23 (1.02)	**-0.28* (-0.39** to **-0.17)**	**NS**		170	78.82 (1.87)	-0.79 (-2.12 to 0.54)	94.8%	
HICs	unexposed to any	3,023	78.15 (0.26)	reference			4,280	78.98 (0.17)	reference		
	<11 years	595	75.44 (0.71)	-0.43 (-1.13 to 0.28)	NS	0.73	159	76.69 (1.03)	-0.21 (-1.70 to 1.29)	76.6%	0.18
	≥11 years	479	76.32 (0.65)	0.08 (-0.70 to 0.86)	NS		76	77.59 (1.49)	-0.78 (-2.91 to 1.35)	87.8%	
LMICs	unexposed to any	4,420	78.56 (0.33)	reference			6,761	80.29 (0.21)	reference		
	<11 years	356	79.21 (1.09)	0.69 (-0.63 to 2.01)	75.4%	0.37	70	78.99 (2.52)	-0.82 (-2.18 to 0.53)	61.0%	0.10
	≥11 years	523	74.88 (1.31)	**-0.29* (-0.41** to **-0.16)**	**NS**		94	79.43 (2.58)	-0.78 (-2.75 to 1.20)	96.3%	
**Fumes (never-smokers)**											
Overall	unexposed to any	3,144	79.78 (0.27)	reference			8,437	80.27 (0.18)	reference		
	<11 years	266	79.80 (0.76)	0.48 (-0.65 to 1.61)	77.9%	0.55	104	78.62 (1.68)	-1.01 (-2.76 to 0.74)	95.8%	0.50
	≥11 years	311	79.66 (1.29)	0.16 (-1.22 to 1.54)	85.0%		114	79.48 (1.94)	0.09 (-1.99 to 2.17)	97.0%	
HICs	unexposed to any	1,126	79.94 (0.33)	reference			2,551	80.20 (0.16)	reference		
	<11 years	163	79.05 (0.68)	0.69 (-0.52 to 1.89)	NS	0.58	56	78.03 (1.06)	-1.47 (-5.19 to 2.25)	98.1%	0.95
	≥11 years	133	79.30 (0.87)	-0.54 (-1.91 to 0.83)	NS		41	80.21 (1.17)	0.43 (-2.54 to 3.41)	97.2%	
LMICs	unexposed to any	2,018	79.75 (0.32)	reference			5,886	80.29 (0.21)	reference		
	<11 years	103	80.25 (1.19)	0.34 (-1.38 to 2.07)	85.1%	0.32	48	79.00 (2.70)	-0.45 (-1.79 to 0.89)	72.3%	0.47
	≥11 years	178	79.78 (1.70)	0.76 (-1.06 to 2.58)	88.9%		73	79.24 (2.60)	-0.31 (-3.69 to 3.07)	97.0%	

HICs: high-income countries; LMICs low- and middle-income countries classified by the World Bank; never-smokers stratification included only participants reporting 'never-smoking'. All Means (SE) were from all 41-site participants; HIC Means (SE) were from 14 high-income site participants; LMIC Means (SE) were from 27 low- and middle-income site participants. The coefficients (β) were adjusted for age (years) and smoking status (never, <20 pack-years, ≥20 pack-years). *p<0.05; NS non-statistically significant (p≥0.05) heterogeneity (I^2); both p<0.05 and I^2=NS in bold.

Table S8. Association of FVC (L) with groups of fume jobs stratified by sex and sites' country economy.

		Men					Women				
		n	**Mean (SE)**	**β (95% CI)**	**I^2(%)**	**p-trend**	***n***	**Mean (SE)**	**β (95% CI)**	**I^2(%)**	**p-trend**
**Fumes (all)**											
Overall	unexposed to any	7,443	3.60 (0.03)	reference			11,041	2.64 (0.04)	reference		
	<11 years	951	3.97 (0.06)	-0.02 (-0.08 to 0.05)	57.6%	0.77	229	3.15 (0.06)	0.11 (-0.01 to 0.23)	89.7%	<0.05
	≥11 years	1,002	3.86 (0.08)	0.00 (-0.06 to 0.06)	54.6%		170	2.97 (0.11)	-0.01 (-0.14 to 0.11)	95.2%	
HICs	unexposed to any	3,023	4.00 (0.04)	reference			4,280	2.84 (0.02)	reference		
	<11 years	595	4.37 (0.05)	-0.04 (-0.14 to 0.05)	56.8%	0.84	159	3.17 (0.05)	0.10 (-0.06 to 0.25)	85.5%	0.82
	≥11 years	479	4.05 (0.05)	0.00 (-0.07 to 0.08)	NS		76	2.96 (0.09)	0.03 (-0.08 to 0.15)	80.5%	
LMICs	unexposed to any	4,420	3.51 (0.05)	reference			6,761	2.58 (0.06)	reference		
	<11 years	356	3.67 (0.09)	0.01 (-0.09 to 0.10)	57.1%	0.98	70	3.13 (0.15)	0.12 (-0.06 to 0.30)	91.5%	<0.05
	≥11 years	523	3.80 (0.10)	-0.01 (-0.09 to 0.07)	63.2%		94	2.98 (0.16)	-0.05 (-0.25 to 0.15)	96.4%	
**Fumes (never-smokers)**											
Overall	unexposed to any	3,144	3.53 (0.04)	reference			8,437	2.60 (0.04)	reference		
	<11 years	266	3.77 (0.07)	-0.05 (-0.27 to 0.18)	96.0%	0.63	104	3.12 (0.11)	0.11 (-0.03 to 0.24)	92.8%	<0.01
	≥11 years	311	3.70 (0.12)	-0.01 (-0.10 to 0.08)	75.8%		114	2.98 (0.13)	0.03 (-0.11 to 0.18)	95.9%	
HICs	unexposed to any	1,126	3.95 (0.07)	reference			2,551	2.69 (0.02)	reference		
	<11 years	163	4.31 (0.11)	-0.07 (-0.21 to 0.08)	63.1%	0.45	56	3.02 (0.10)	0.08 (-0.11 to 0.28)	92.5%	<0.05
	≥11 years	133	4.11 (0.07)	0.06 (-0.11 to 0.22)	55.5%		41	2.97 (0.12)	0.13* (0.01 to 0.26)	85.0%	
LMICs	unexposed to any	2,018	3.45 (0.04)	reference			5,886	2.58 (0.06)	reference		
	<11 years	103	3.46 (0.08)	-0.04 (-0.39 to 0.30)	97.5%	0.96	48	3.18 (0.15)	0.13 (-0.06 to 0.31)	90.9%	<0.01
	≥11 years	178	3.56 (0.16)	-0.03 (-0.14 to 0.07)	80.6%		73	2.99 (0.17)	-0.08 (-0.27 to 0.11)	95.6%	

HICs: high-income countries; LMICs low- and middle-income countries classified by the World Bank; never-smokers stratification included only participants reporting 'never-smoking'. All Means (SE) were from all 41-site participants; HIC Means (SE) were from 14 high-income site participants; LMIC Means (SE) were from 27 low- and middle-income site participants. The coefficients (β) were adjusted for age (years), height (cm) and smoking status (never, <20 pack-years, ≥20 pack-years). *p<0.05; NS non-statistically significant (p≥0.05) heterogeneity (I^2); both p<0.05 and I^2=NS in bold.
