# Supplementary material for: Association of respiratory symptoms and lung function with occupation in the multinational Burden of Obstructive Lung Disease (BOLD) study
Source: Eur Respir J. 2023 Jan 12;61(1):2200469. doi: 10.1183/13993003.00469-2022 (PMC9834632; doi:10.1183/13993003.00469-2022)
Supplement: Supplementary file 2 [file ERJ-00469-2022.Shareable.pdf]

EUROPEAN RESPIRATORY JOURNAL
ORIGINAL RESEARCH ARTICLE
J. RATANACHINA ET AL.

SHAREABLE PDF

Association of respiratory symptoms and lung function with occupation in the multinational Burden of Obstructive Lung Disease (BOLD) study

Jate Ratanachina[1,2,3], Andre F.S. Amaral[1], Sara De Matteis[1,4], Herve Lawin[5], Kevin Mortimer[6,7], Daniel O. Obaseki[8], Imed Harrabi[9], Meriam Denguezli[9], Emiel F.M. Wouters[10,11], Christer Janson[12], Rune Nielsen[13], Amund Gulsvik[13], Hamid Hacene Cherkaski[14], Filip Mejza[15], Padukudru Anand Mahesh[16], Asma Elsony[17], Rana Ahmed[17], Wan Tan[18], Li Cher Loh[19], Abdul Rashid[19], Michael Studnicka[20], Asaad A. Nafees[21], Terence Seemungal[22], Althea Aquart-Stewart[23], Mohammed Al Ghobain[24], Jinping Zheng[25], Sanjay Juvekar[26], Sundeep Salvi[27], Rain Jogi[28], David Mannino[29], Thorarinn Gislason[30,31], A. Sonia Buist[32], Paul Cullinan[1] and Peter Burney[1], for the BOLD Collaborative Research Group[1]

[1]National Heart and Lung Institute, Imperial College London, London, UK. [2]Dept of Preventive and Social Medicine, King Chulalongkorn Memorial Hospital, The Thai Red Cross Society, Bangkok, Thailand. [3]Dept of Preventive and Social Medicine, Faculty of Medicine, Chulalongkorn University, Bangkok, Thailand. [4]Dept of Medical Sciences and Public Health, University of Cagliari, Cagliari, Italy. [5]Unit of Teaching and Research in Occupational and Environmental Health, Cotonou, Benin. [6]University of Cambridge, Cambridge, UK. [7]Liverpool University Hospitals NHS Foundation Trust, Liverpool, UK. [8]Obafemi Awolowo University, Ile-Ife, Nigeria. [9]Faculte de Medecine, Sousse, Tunisia. [10]Ludwig Boltzmann Institute for Lung Health, Vienna, Austria. [11]Maastricht University Medical Center, Maastricht, The Netherlands. [12]Respiratory, Allergy and Sleep Research, Dept of Medical Sciences, Uppsala University, Uppsala, Sweden. [13]Dept of Clinical Science, Faculty of Medicine, University of Bergen, Bergen, Norway. [14]Dept of Pneumology, Faculty of Medicine and CHU Annaba, Annaba, Algeria. [15]Center for Evidence Based Medicine, 2nd Department of Internal Medicine, Jagiellonian University Medical College, Kraków, Poland. [16]JSS Medical College, JSSAHER, Mysuru, India. [17]The Epidemiological Laboratory, Khartoum, Sudan. [18]Centre for Heart Lung Innovation, University of British Columbia, Vancouver, BC, Canada. [19]Royal College of Surgeons in Ireland and University College Dublin Malaysia Campus, Penang, Malaysia. [20]Dept of Pulmonary Medicine, Paracelsus Medical University, Salzburg, Austria. [21]Aga Khan University, Karachi, Pakistan. [22]University of the West Indies, St Augustine, Trinidad and Tobago. [23]University of the West Indies, Kingston, Jamaica. [24]College of Medicine, King Saud bin Abdulaziz University for Health Sciences, King Abdulaziz Medical City in Riyadh, Saudi Arabia. [25]State Key Laboratory of Respiratory Disease, National Clinical Research Center for Respiratory Diseases, Guangzhou Institute of Respiratory Health, First Affiliated Hospital of Guangzhou Medical College, Guangzhou, China. [26]Vadu Rural Health Program, KEM Hospital Research Centre Pune, Pune, India. [27]Pulmocare Research and Education Foundation, Pune, India. [28]Lung Clinic, Tartu University Hospital, Tartu, Estonia. [29]University of Kentucky, Lexington, KY, USA. [30]Dept of Sleep, Landspitali University Hospital, Reykjavik, Iceland. [31]Faculty of Medicine, University of Iceland, Reykjavik, Iceland. [32]Oregon Health and Science University, Portland, OR, USA.

Corresponding author: Andre Amaral (a.amaral@imperial.ac.uk)

Check for updates

Shareable abstract (@ERSpublications)
Occupational exposures associate with more respiratory symptoms but do not appear to be major determinants of differences in lung function. https://bit.ly/3zFjalu

Cite this article as: Ratanachina J, Amaral AFS, De Matteis S, *et al*. Association of respiratory symptoms and lung function with occupation in the multinational Burden of Obstructive Lung Disease (BOLD) study. *Eur Respir J* 2022; 0: 2200469 [DOI: 10.1183/13993003.00469-2022].

This single-page version can be shared freely online.

Copyright ©The authors 2022. For reproduction rights and permissions contact permissions@ersnet.org

Received: 13 March 2022
Accepted: 2 Aug 2022

Abstract

Background Chronic obstructive pulmonary disease has been associated with exposures in the workplace. We aimed to assess the association of respiratory symptoms and lung function with occupation in the Burden of Obstructive Lung Disease study.

Methods We analysed cross-sectional data from 28 823 adults (⩾40 years) in 34 countries. We considered 11 occupations and grouped them by likelihood of exposure to organic dusts, inorganic dusts and fumes. The association of chronic cough, chronic phlegm, wheeze, dyspnoea, forced vital capacity (FVC) and forced expiratory volume in 1 s (FEV_1)/FVC with occupation was assessed, per study site, using

multivariable regression. These estimates were then meta-analysed. Sensitivity analyses explored differences between sexes and gross national income.

Results Overall, working in settings with potentially high exposure to dusts or fumes was associated with respiratory symptoms but not lung function differences. The most common occupation was farming. Compared to people not working in any of the 11 considered occupations, those who were farmers for ⩾20 years were more likely to have chronic cough (OR 1.52, 95% CI 1.19–1.94), wheeze (OR 1.37, 95% CI 1.16–1.63) and dyspnoea (OR 1.83, 95% CI 1.53–2.20), but not lower FVC (β=0.02 L, 95% CI −0.02–0.06 L) or lower FEV_1/FVC (β=0.04%, 95% CI −0.49–0.58%). Some findings differed by sex and gross national income.

Conclusion At a population level, the occupational exposures considered in this study do not appear to be major determinants of differences in lung function, although they are associated with more respiratory symptoms. Because not all work settings were included in this study, respiratory surveillance should still be encouraged among high-risk dusty and fume job workers, especially in low- and middle-income countries.
